# Protective effect of the Impella on the left ventricular function after acute broad anterior wall ST elevation myocardial infarctions with cardiogenic shock: cardiovascular magnetic resonance imaging strain analysis

**DOI:** 10.1186/s12872-022-02632-7

**Published:** 2022-04-28

**Authors:** Daisuke Fukamachi, Akimasa Yamada, Akihito Ohgaku, Yutaka Koyama, Hidesato Fujito, Riku Arai, Yasunari Ebuchi, Suguru Migita, Tomoyuki Morikawa, Masaki Monden, Norio Takei, Takehiro Tamaki, Keisuke Kojima, Naotaka Akutsu, Nobuhiro Murata, Yuki Saito, Daisuke Kitano, Mitsumasa Sudo, Yasuo Okumura

**Affiliations:** Division of Cardiology, Department of Medicine, Nihon University School of Medicine, Ohyaguchi-kamicho, Itabashi-ku, Tokyo, 173-8610 Japan

**Keywords:** Anterior ST-elevation myocardial infarction, Impella, Cardiac magnetic resonance imaging

## Abstract

**Background:**

The clinical efficacy of the Impella for high-risk percutaneous coronary intervention (PCI) and cardiogenic shock remains under debate. We thus sought to investigate the protective effects on the heart with the Impella’s early use pre-PCI using cardiac magnetic resonance imaging (CMRI).

**Methods:**

We retrospectively evaluated the difference in the subacute phase CMR imaging results (19 ± 9 days after admission) between patients undergoing an Impella (n = 7) or not (non-Impella group: n = 18 [12 intra-aortic balloon pumps (1 plus veno-arterial extracorporeal membrane oxygenation) and 6 no mechanical circulation systems]) in broad anterior ST-elevation myocardial infarction (STEMI) cases. A mechanical circulation system was implanted pre-PCI.

**Results:**

No differences were found in the door-to-balloon time, peak creatine kinase, and hospital admission days between the Impella and non-Impella groups; however, the CMRI-derived left ventricular ejection fraction was significantly greater (45 ± 13% vs. 34 ± 7.6%, *P* = 0.034) and end-diastolic and systolic volumes smaller in the Impella group (149 ± 29 vs. 187 ± 41 mL, *P* = 0.006: 80 ± 29 vs. 121 ± 40 mL, *P* = 0.012). Although the global longitudinal peak strain did not differ, the global radial (GRS) and circumferential peak strain (GCS) were significantly higher in the IMPELLA than non-IMPELLA group. Greater systolic and diastolic strain rates (SRs) in the Impella than non-Impella group were observed in non-infarcted rather than infarcted areas.

**Conclusions:**

Early implantation of an Impella before PCIs for STEMIs sub-acutely prevented cardiac dysfunction through preserving the GRS, GCS, and systolic and diastolic SRs in the remote myocardium. This study provided mechanistic insight into understanding the usefulness of the Impella to prevent future heart failure.

**Supplementary Information:**

The online version contains supplementary material available at 10.1186/s12872-022-02632-7.

## Background

The survival rate from an acute myocardial infarction (AMI) has generally improved due to the technological advancement in Japan [1]; however, an ST-elevation myocardial infarction (STEMI) with cardiogenic shock has a high mortality rate, even after shortening the door-to-balloon time (DTBT) [2]. Additionally, heart failure associated with a STEMI tends to increase due to the development of left ventricular (LV) remodeling; thereafter, the prognosis worsens [3].

Previously, the main treatment strategy for cardiogenic shock in those with an AMI was to maintain the hemodynamics using mechanical circulatory support provided by intra-aortic balloon pumping (IABP) and veno-arterial extracorporeal membrane oxygenation (VA-ECMO**)**. Earlier studies demonstrated that IABP reduces the afterload and improves the coronary blood flow [4]; however, although IABP stabilizes the hemodynamics, the afterload reduction was ineffective. Thus, IABP does not improve the 30-day survival in STEMI patients with cardiogenic shock [5]. While VA-ECMO is a powerful technique that assists with the systemic circulation, it increases the LV load and afterload.

Under these circumstances, the Impella 2.5 or 5.0 (Abiomed, Danvers, MA, USA), a percutaneous LV assist device for drug-resistant cardiogenic shock, has been used since 2017 in Japan; the Impella CP (Abiomed), designed to provide a higher level of support than the Impella 2.5, has been available since 2019. By drawing arterial blood from the left ventricle to the aorta, it is possible to directly unload the left ventricle. Direct LV unloading lowers the LV end-diastolic pressure, improving the blood gas oxygenation and systemic perfusion [6]. Several reports have reported that the use of the Impella in the acute phase increases the cardiac output, improving the coronary blood flow [7, 8]. Unexpectedly, the IMPRESS in Severe Shock trial, the first randomized pilot trial to compare the efficacy and safety of the Impella CP versus IABP, did not show any 3-day or 6-month mortality in the 48 patients (24 per group) [9]; that was possibly due to the fact that the Impella increases bleeding events, worsening the renal function and cerebral infarctions, and causes death during coronary treatment [10]. A major concern was that the Impella was placed “after” revascularization in 80% of the study patients; an earlier study demonstrated an improved survival in patients who received an Impella pre-, rather than post-percutaneous coronary intervention (PCI) [11]. Further, over 90% of participants were resuscitated cardiac arrest patients, potentially decreasing the beneficial effects of the Impella.

We, therefore, hypothesized that the Impella would provide some beneficial effects of LV remodeling for patients who received the device before the PCI and had not suffered from a cardiac arrest. We retrospectively evaluated the data regarding the LV function by cardiac magnetic resonance imaging (CMRI) in patients with anterior STEMIs who had undergone an Impella placement just before the PCI; that was compared with patients without an Impella.

## Methods

### Study population

**Fig. 1:**
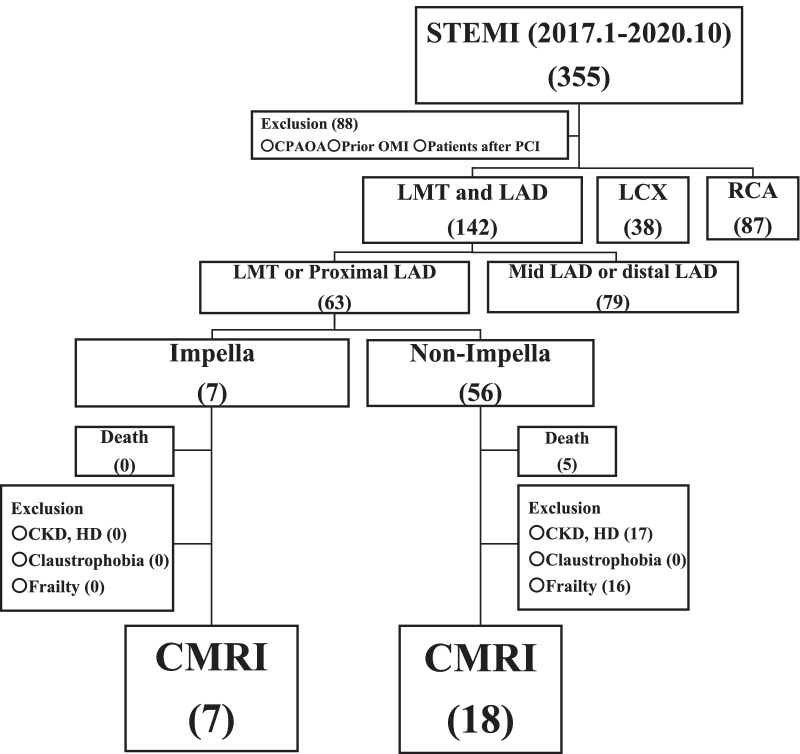
Flow chart of the study participants. *CKD* chronic kidney disease, *CMRI* cardiac magnetic resonance imaging, *CPAOA* cardiopulmonary arrest on arrival, *HD* hemodialysis, *LAD* left anterior descending artery, *LCX* left circumflex artery, *LMT* left main trunk, *OMI* old myocardial infarction, *PCI* percutaneous coronary intervention, *RCA* right coronary artery, *STEMI* ST-elevation myocardial infarction

In order to investigate the protective effects on the heart by the early use of an Impella before the PCI using CMRI, this study retrospectively enrolled 63 consecutive patients with broad anterior STEMIs who were admitted to our hospital between January 2017 and October 2020. The inclusion criteria were having undergone a protected PCI for the first broad anterior STEMI, followed by CMRI before discharge. The exclusion criteria were patients with a cardiopulmonary arrest on arrival, history of an old myocardial infarction or PCI, impaired consciousness, and those who did not undergo a CMRI due to renal failure including dialysis, claustrophobia, or frailty. In all patients, the culprit lesion was the proximal left anterior descending (LAD) or left main trunk. A STEMI is universally defined as a myocardial infarction, where ST-elevation refers to two or more leads. In the V2-3 leads, the ST-elevation was ≥ 2.0 mm in men > 40 years, ≥ 2.5 mm in men < 40 years, and ≥ 1.5 mm in women regardless of their age; an ST rise was ≥ 1.0 mm. Figure 1 shows the flowchart of the study participants. Among 63 broad anterior STEMI patients, an Impella was used in 7 but not in 56. None of the 7 Impella patients were excluded, but among the 51 non-Impella patients, 33 fulfilled the exclusion criteria. As a result, 7 patients in the Impella group and 18 in the non-Impella group were selected as the study participants. All patients had consented, by the opt-out method, to use their data for the study purposes. The study protocol was reviewed and approved by the Nihon University Itabashi Hospital, Clinical Research Judging Committee (RK-200714-10) and was in accordance with the ethical standards of the institutional research committee and 1964 Declaration of Helsinki.

### Data collection

The patient characteristics and follow-up data were retrospectively obtained from our hospital records. The patient background at the time of the PCI for ACS was anonymized and extracted; the information included the age, sex, age, body mass index (BMI), smoking history, comorbidities (hypertension, diabetes, or dyslipidemia), hemodynamic variables (heart rate and systolic and diastolic blood pressure), laboratory tests (hemoglobin concentration, lactate, and estimated glomerular filtration rate [eGFR]), and transthoracic echocardiographic parameters (LV ejection fraction [LVEF], LV end-diastolic volume [LVEDV], and LV end-systolic volume [LVESV]) obtained at the time of the emergency CAG. The N-terminal pro-brain natriuretic peptide (NT-proBNP) levels at discharge were used for the analysis. Cardiogenic shock was defined by the following three criteria: (1) an SBP < 90 mm Hg with appropriate fluid resuscitation and clinical and laboratory evidence of end‐organ damage, (2) clinical: old extremities, oliguria, altered mental status, and narrow pulse pressure, and (3) laboratory: metabolic acidosis, and elevated serum lactate and serum creatinine levels [12].

### Implantation of mechanical circulatory support and post-MI medical treatments

The choice between a mechanical circulatory support device (Impella, IABP, VA-ECMO) or none was made at the physicians’ discretion, but the indication of a mechanical circulatory support device was generally decided according to the following criteria: (1) before 2018, the use of an Impella was not available in Japan, therefore, IABP was used in cardiogenic shock, multivessel disease, and/or hemodynamically unstable cases during the PCI, and (2) from 2018, an Impella was used in cardiogenic shock cases immediately after admission, but IABP was used for multivessel disease or hemodynamically unstable cases during the PCI. During the study period, the use of VA-ECMO was decided when cardiogenic shock persisted despite the use of IABP or an Impella. In all cases with an IABP or Impella, both the IABP and Impella were placed just before the PCI. The Impella 2.5 or CP was used, while the 5.0 model was not. The patient entered the coronary care unit after the PCI for intensive care. The IABP and Impella were removed after the heart failure condition stabilized following starting a β-blocker and renin-angiotensin system inhibitor. Thereafter, they were transferred to a general ward to undergo cardiac rehabilitation; the amount of cardioprotective agents was carefully increased, while all pressor agents, such as the dobutamine infusion, were tapered.

### Cardiac magnetic resonance imaging

In our hospital, CMRI was generally planned in patients with a broad anterior STEMI for the follow-up medical management if the patients were tolerant to undergoing CMRI. All patients underwent CMRI before discharge if they had a stable condition, which was defined as no evidence of congestion or pneumonia on the chest-Xray and were capable of walking with stable vital signs. The images were carried out using a 1.5-T scanner (Ingenia; Philips Healthcare, Eindhoven, Netherlands) with retrospective electrocardiographic gating and an ads Torso coil. An LV analysis was performed offline with commercially available software (cvi42, version 4.1.8, Circle Cardiovascular Imaging, Calgary, Canada). The CMRI protocol included a standard steady-state free precession (SSFP) cine and late gadolinium enhancement (LGE) MRI. Standard SSFP cine images covered the entire LV using short-axis slices and 2-, 3-, and 4-chamber views (temporal resolution of < 40 ms). The LVEDV, LVESV, stroke volume, LVEF, and LV mass were calculated as previously described [13].

**Fig. 2:**
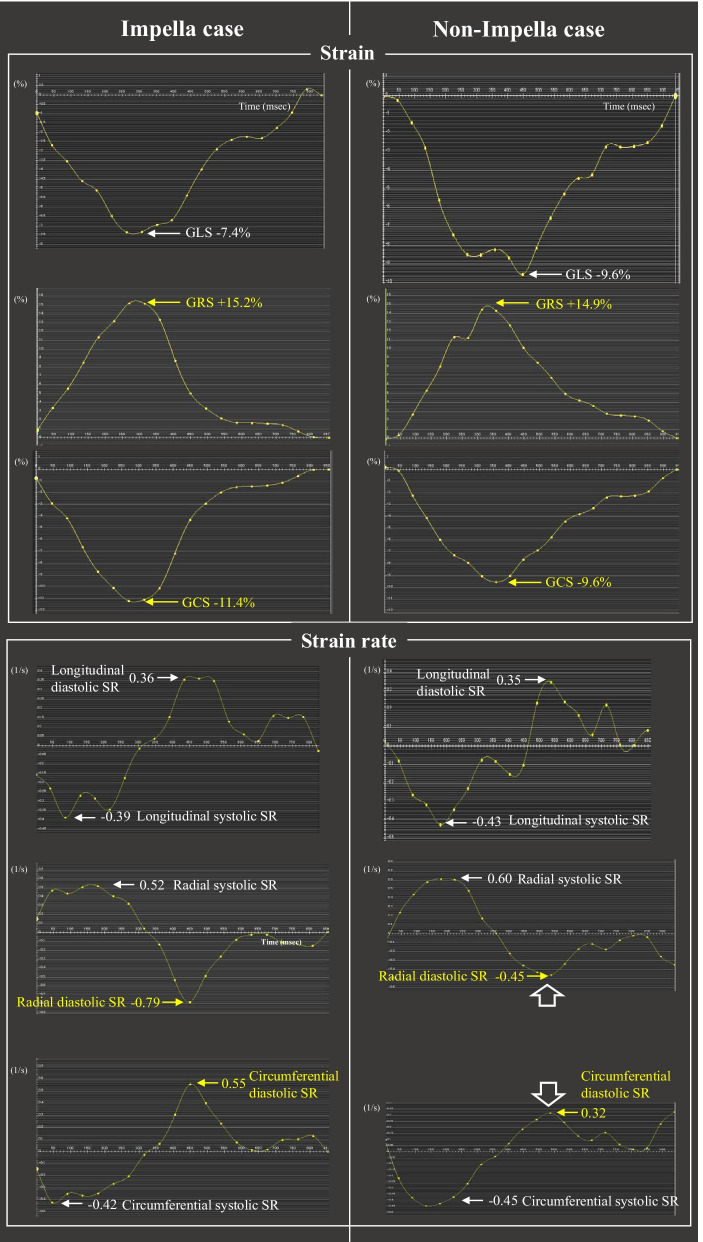
Representative CMRI strains and SR images in the Impella and non-Impella cases. CMRI strains: global longitudinal, radial, and circumferential peak strains (GLS, GRS, and GCS); SR images: longitudinal, radial, and circumferential systolic and diastolic SRs. Note that the magnitudes of the GRS and GCS and radial and circumferential diastolic SRs are higher in the Impella group than non-Impella group, but higher values of those parameters are not observed for the longitudinal axis. *CMRI* cardiac magnetic resonance imaging, *SR* strain rate

For the LV strain analysis, the endo- and epicardial borders were semi-automatically delineated at end-diastole in the short- and long-axis cines (excluding the papillary muscles) and automatically propagated to all slices throughout the cardiac cycle. Short-axis cines were tracked to derive the radial and circumferential strain, while 2-, 3, and 4-chamber-view cines were tracked to derive the longitudinal strain [13]. Based on the 16-segment model, the software algorithm calculated the 2D peak strains (longitudinal, radial, and circumferential directions) by averaging the corresponding peak values of the segments. In all analyses, the strain was defined as the average of the peaks of the global, infarcted, and non-infarcted strain curves; the systolic strain rate (SR) was the average of the peaks of the global, infarcted, and non-infarcted SR curves during systole, and the diastolic SR was the peak during diastole (Fig. 2).

The LGE imaging was acquired with a T1-weighted inversion recovery gradient-echo sequence 15 min after a contrast injection (0.15 mmol/kg; Gd-BTDO3A; Gadovist, Bayer Japan, Tokyo, Japan). The images were acquired on three long-axis slices (2-, 3-, and 4-chamber) and a stack of the short-axis slices covering the entire LV was obtained [13]. The infarct size was quantified by the full-width with the half-maximum method [14]. The LV myocardium was divided into infarcted and non-infarcted areas. Based on the segmental coronary artery distribution model from the American Society of Echocardiography and European Association of Cardiovascular Imaging guidelines [15], the infarcted area was defined as the proximal LAD perfusion territory, including segments 1–2, 7–8, and 13–16, whereas the non-infarcted area was defined as the remaining LV myocardium [14].

### Post-discharge follow-up

Each patient was followed up at our outpatient cardiology clinic around 3, 6, 9, and 12 months.

### Study assessments

The CMRI at the assessment endpoint included measurements of the LVEF, LVEDV, LVESV, LV mass, and infarcted size. The strain analysis involved the longitudinal, radial, and circumferential peak, systolic, and diastolic SRs in the global, infarcted, and non-infarcted areas. We also ascertained the clinical events including hospitalizations due to heart failure, all-cause death, non-fatal myocardial infarctions, strokes, stent thromboses, and major bleeding via the medical records.

### Statistical analysis

The values are shown as the mean ± SD, median, and interquartile ranges and the number and percentages. The continuous variables between the groups were analyzed using the Mann–Whitney U-test; differences in the categorical variables were tested using the chi-square test or Fisher’s exact text. The comparisons among the continuous variables between the Impella and non-Impella groups with and without cardiogenic shock were analyzed by the Kruskal–Wallis test, followed by a Bonferroni corrected Mann–Whitney U post-hoc analysis. For the categorical valuables, a chi-square test was used. All analyses were performed using SPSS version 19.0 software (SPSS Inc., Chicago, IL, USA); a *P* < 0.05 was considered statistically significant.

## Results

### Baseline characteristics between the Impella and non-Impella groups

An Impella was used for mechanical support in 7 patients (2.5: 5 patients; CP: 2 patient) because they had experienced cardiogenic shock, but 1 of them needed a VA-ECMO due to sustained cardiogenic shock. Among the 18 patients in the non-Impella group, 12 received an IABP due to cardiogenic shock (n = 4: one patient received a VA-ECMO due to sustained cardiogenic shock despite the use of IABP), were hemodynamical unstable during the PCI (n = 7), or had multivessel disease (n = 1), and the remaining 6 had no mechanical circulatory support because they were relatively hemodynamically stable. As a result, in the Impella group, all patients had cardiogenic shock, while in the non-Impella group, 28% (5/18) of patients had cardiogenic shock. There were no differences in the age (67 ± 10 vs. 59 ± 13 years, *P* = 0.13), a male sex (86 vs. 94%, *P* = 0.47), nor the prevalence of hypertension, diabetes, and smoking between the Impella and non-Impella groups, but the Impella group had a lower BMI (21 ± 1.5 vs. 25 ± 3.7 kg/m^2^, *P* = 0.039) and higher rate of dyslipidemia (100 vs. 44%, *P* = 0.013). On admission, the systolic blood pressure was significantly lower in the Impella group (97 ± 25 vs. 121 ± 35 mmHg, *P* = 0.034), but the difference in the minimum systolic blood pressure from admission to the PCI was reduced between the two groups (91 ± 20 vs. 105 ± 31 mmHg, *P* = 0.06). No difference was observed in the lactic acid level (2.9 ± 1.4 vs. 2.2 ± 1.0 mmol/l, *P* = 0.24), eGFR (65 ± 16 vs. 74 ± 20 mL/min/1.73 m^2^, *P* = 0.33), and TIMI 0 flow cases of the LAD on angiography before the PCI (57 vs. 61%, *P* = 0.60). The DTBT did not differ between the two groups (60 ± 16 vs. 55 ± 24 min, *P* = 0.58). There were no differences in the transthoracic echocardiographic LVEF (49 ± 11 vs. 48 ± 7.3%, *P* = 0.79), LVEDV (107 ± 15 vs. 118 ± 32 ml, *P* = 0.38), LVESV (50 ± 16 vs. 57 ± 20 ml, *P* = 0.36), E (73 ± 12 vs. 64 ± 21 cm/s, *P* = 0.20), A (73 ± 21 vs. 67 ± 23 cm/s, *P* = 0.46), and E/A (1.1 ± 0.5 vs. 1.1 ± 0.6, *P* = 0.75) on admission between the two groups. The Impella was in place for 4.0 ± 1.7 days (Table 1).

**Table 1:** Baseline characteristics between patients with and without an Impella

	Impella (n = 7)	Non-Impella (n = 18)	*P* value
Age, years	67 ± 10	59 ± 13	0.13
Male gender	6 (86)	17 (94)	0.47
BMI (m^2^/kg)	21 ± 1.5	25 ± 3.7	0.039
Hemodynamic variables on admission			
Heart rate (beats/min)	85 ± 18	88 ± 18	0.66
Systolic blood pressure (mmHg)	97 ± 25	121 ± 35	0.034
Diastolic blood pressure (mmHg)	77 ± 19	84 ± 27	0.36
Minimum systolic blood pressure from admission to PCI	91 ± 20	105 ± 31	0.06
Cardiogenic shock	7 (100)	5 (28)	0.010
History or comorbidities			
Current smoking	1 (17)	9 (53)	0.12
Hypertension	5 (71)	12 (67)	0.82
Diabetes mellitus	1 (14)	5 (28)	0.44
Dyslipidaemia	7 (100)	8 (44)	0.013
Blood values on admission			
Lactate (mmol/l)	2.9 ± 1.4	2.2 ± 1.0	0.24
Hb (mg/dl)	13.4 ± 1.6	14.8 ± 1.2	0.06
eGFR (mL/min/1.73 m^2^)	65 ± 16	74 ± 20	0.33
Blood values at discharge			
NT-proBNP at discharge (pg/ml)	922 (130, 1643)	1645 (559, 2189)	0.32
Support device			
Mechanical ventilation	2 (29)	3 (17)	0.50
IABP	0 (0)	12 (67)	0.004
VA-ECMO	1 (14)	1 (6)	0.46
Anterior STEMI	7 (100)	18 (100)	–
Infarct-related artery			
Left main stem	1 (17)	0 (0)	0.74
Left anterior descending	6 (83)	18 (100)	
Multivessel disease	1 (17)	1(6)	0.46
Stent placement			
Drug-eluting stent	6 (100)	17 (100)	
Number of DES stents	1.2 ± 0.4	1.2 ± 0.4	0.96
Initial TIMI flow 0	4 (57)	11 (61)	0.60
Final TIMI flow 3	7 (100)	17 (94)	0.72
Max CK (IU/L)	7058 ± 4994	6856 ± 4673	0.96
	5964 (2262, 11,273)	5670 (4317, 8655)	
Max CK MB (IU/L)	527 ± 355	539 ± 393	0.99
	542 (181, 904)	395 (281, 629)	
Door to balloon time (min)	60 ± 16	55 ± 24	0.58
Echo parameter on admission			
LVEF (%)	49 ± 11	48 ± 7.3	0.79
LVEDV (mL)	107 ± 15	118 ± 32	0.53
LVESV (mL)	50 ± 16	57 ± 20	0.36
E (cm/s)	73 ± 12	64 ± 21	0.20
A (cm/s)	73 ± 21	67 ± 23	0.46
E/A	1.1 ± 0.5	1.1 ± 0.6	0.75
Duration of IMPELLA support (days)	4.0 ± 1.7	–	–
Medications at discharge			
DAPT	7 (100)	18 (100)	–
RAS inhibitor	7 (100)	18 (100)	–
Beta blocker	7 (100)	17 (94)	0.72
Statin	7 (100)	18 (100)	–
Days of coronary care unit (days)	7.4 ± 3.7	6.4 ± 4.0	0.56
Days of hospital admission (days)	27 ± 8	26 ± 8	0.65
Days of CMRI after admission (days)	16 ± 7	20 ± 10	0.36

Values are shown as the number (%), mean ± SD, or median (interquartile ranges)

*BMI* body mass index, *CMRI* cardiac magnetic resonance image, *CK* creatinine kinase, *DES* drug eluting stent, *DAPT* dual antiplatelet therapy, *eGFR* estimated glomerular filtration rate, *Hb* haemoglobin, *IABP* intra-aortic balloon pumping, *LVEF* left ventricular ejection fraction, *LVEDV* left ventricular end-diastole volume, *LVESV* left ventricular end-systolic volume, *NT-pro BNP* n-terminal pro brain natriuretic peptide, *PCI* percutaneous coronary intervention, *RAS* renin angiotensin system, *STEMI* ST-elevation myocardial infarction, *TIMI* thrombolysis in myocardial infarction, *VA-ECMO* veno-arterial extracorporeal membrane oxygenation

*P* values are determined by a Mann–Whitney U test, chi-square test, or Fisher exact test

### CMRI assessment between the Impella and non-Impella groups

**Fig. 3:**
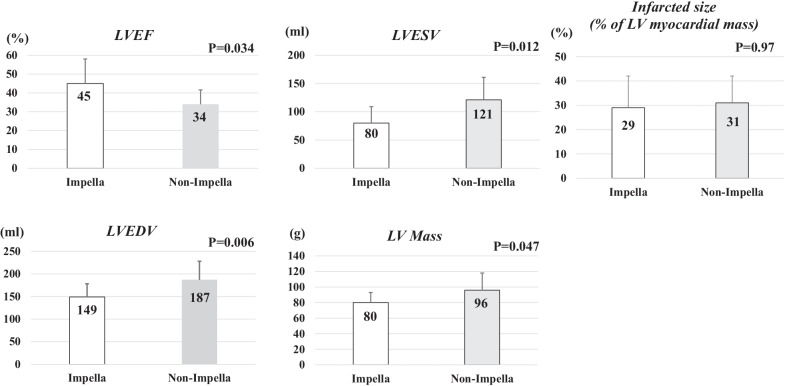
Comparison of the LVESV, LVEDV, EF, LV mass, and infarcted size between the Impella (white bar) and non-Impella groups (grey bar). *EF* ejection fraction, *LVEDV* left ventricular end-diastole volume, *LVESV* left ventricular end-systolic volume, *LV* left ventricular. *P* values are analyzed by Mann–Whitney U text

CMRI was performed 19 ± 8 (range 6–40) days after admission (Impella: 16 ± 7 [range 7–23] days; non-Impella: 20 ± 10 [range 6–40] days, *P* = 0.36). The LVEF was significantly greater (45 ± 13 vs. 34 ± 7.6%, *P* = 0.034) and LV mass (80 ± 13 vs. 96 ± 22 g, *P* = 0.047), LVEDV (149 ± 29 vs. 187 ± 41 mL, *P* = 0.006), and LVESV (80 ± 29 vs. 121 ± 40 mL, *P* = 0.012) smaller in the Impella than non-Impella group; no difference was observed in the infarcted size of the LV myocardial mass (29 ± 13 vs. 31 ± 11%, *P* = 0.97; Fig. 3).

**Fig. 4:**
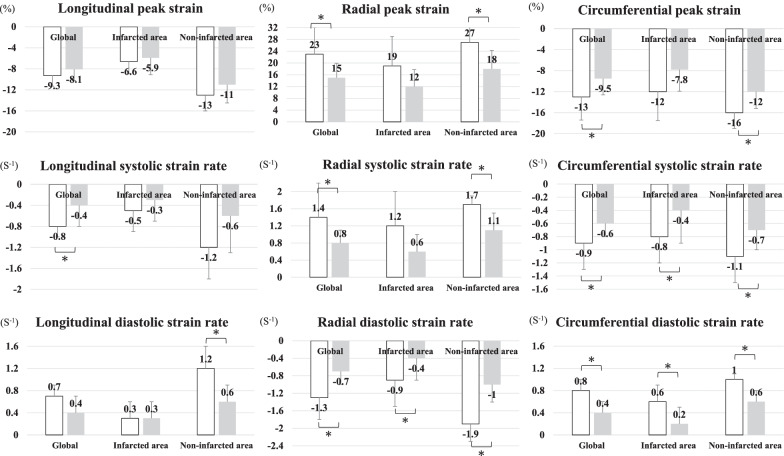
CMRI strain parameters between the Impella group (white bar) and non-Impella group (grey bar). *CMRI* cardiac magnetic resonance imaging. *P < 0.05 by a Mann–Whitney U text

Representative CMRI results of the two groups are shown in Fig. 2; there was no significant difference in the global longitudinal peak strain (GLS) between the Impella and non-Impella groups (GLS: − 9.3 ± 2.0 vs. − 8.1 ± 2.1, *P* = 0.43), but the global radial (GRS) and circumferential peak strain (GCS) were significantly higher in the Impella group than non-Impella group (GRS: 23 ± 9.1 vs. 15 ± 4.9, *P* = 0.044; GCS: − 13 ± 4.4 vs. − 9.5 ± 3.1, *P* = 0.024). The global radial and circumferential systolic and diastolic SRs were significantly higher in the Impella group than non-Impella group, but only the global longitudinal systolic SR was significantly higher. Regarding the infarcted and non-infarcted areas, the increases in the peak strain and systolic and diastolic SRs in the Impella were greater in the radial and circumferential axes as compared to the longitudinal axis than in the non-Impella group, and were also greater in the non-infarcted area than in the infarcted area (Fig. 4, Table 2).

**Table 2:** CMRI strain parameters between the patients with and without an Impella

CMRI strain parameters	Impella (n = 7)	Non-Impella (n = 18)	*P* value
LVEF (%)	45 ± 13	34 ± 7.6	0.034
LVEDV (mL)	149 ± 29	187 ± 41	0.006
LVESV (mL)	80 ± 29	121 ± 40	0.012
LV Mass (g)	80 ± 13	96 ± 22	0.047
Infarcted size (% of LV myocardial mass)	29 ± 13	31 ± 11	0.97
*LV longitudinal*			
Global			
Peak strain (%)	− 9.3 ± 2.0	− 8.1 ± 2.1	0.43
Systolic strain rate (1/s)	− 0.8 ± 0.2	− 0.4 ± 0.4	0.020
Diastolic strain rate (1/s)	0.7 ± 0.2	0.4 ± 0.3	0.12
Infarcted area			
Peak strain (%)	− 6.6 ± 2.3	− 5.9 ± 3.2	0.76
Systolic strain rate (1/s)	− 0.5 ± 0.4	− 0.3 ± 0.4	0.29
Diastolic strain rate (1/s)	0.3 ± 0.3	0.3 ± 0.3	0.81
Non-infarcted area			
Peak strain (%)	− 13 ± 3.0	− 11 ± 3.5	0.32
Systolic strain rate (1/s)	− 1.2 ± 0.5	− 0.6 ± 0.7	0.12
Diastolic strain rate (1/s)	1.2 ± 0.4	0.6 ± 0.5	0.024
*LV radial*			
Global			
Peak strain (%)	23 ± 9.1	15 ± 4.9	0.044
Systolic strain rate (1/s)	1.4 ± 0.8	0.8 ± 0.3	0.024
Diastolic strain rate (1/s)	− 1.3 ± 0.5	− 0.7 ± 0.3	0.006
Infarcted area			
Peak strain (%)	19 ± 10	12 ± 5.8	0.12
Systolic strain rate (1/s)	1.2 ± 0.8	0.6 ± 0.4	0.052
Diastolic strain rate (1/s)	− 0.9 ± 0.6	− 0.4 ± 0.5	0.074
Non-infarcted area			
Peak strain (%)	27 ± 8.2	18 ± 6.3	0.020
Systolic strain rate (1/s)	1.7 ± 0.7	1.1 ± 0.4	0.024
Diastolic strain rate (1/s)	− 1.9 ± 0.4	− 1.0 ± 0.3	< 0.001
*LV circumferential*			
Global			
Peak strain (%)	− 13 ± 4.4	− 9.5 ± 3.1	0.024
Systolic strain rate (1/s)	− 0.9 ± 0.4	− 0.6 ± 0.2	0.044
Diastolic strain rate (1/s)	0.8 ± 0.2	0.4 ± 0.2	0.001
Infarcted area			
Peak strain (%)	− 12 ± 5.5	− 7.8 ± 4.1	0.16
Systolic strain rate (1/s)	− 0.8 ± 0.4	− 0.4 ± 0.5	0.10
Diastolic strain rate (1/s)	0.6 ± 0.3	0.2 ± 0.3	0.024
Non-infarcted area			
Peak strain (%)	− 16 ± 3.0	− 12 ± 3.2	0.010
Systolic strain rate (1/s)	− 1.1 ± 0.4	− 0.7 ± 0.2	0.030
Diastolic strain rate (1/s)	1.0 ± 0.1	0.6 ± 0.2	< 0.001

*CMRI* cardiac magnetic resonance image, *LV* left ventricular, *LVEF* left ventricular ejection fraction, *LVEDV* left ventricular end-diastole volume, *LVESV* left ventricular end-systolic volume

*P* values are determined by a Mann–Whitney U test

### Clinical outcomes

No difference was found in the extent of the infarction as evidenced by the maximum creatine kinase (7058 ± 4994 vs. 6856 ± 4673 IU/L, *P* = 0.96) and creatine kinase-MB (527 ± 355 vs. 539 ± 393 IU/L, *P* = 0.99) between the two groups, nor in the length of the hospital admission (27 ± 8 vs. 26 ± 8 days, *P* = 0.65) and NT-proBNP level at discharge (922 [130, 1643] vs. 1645 [559, 2189] pg/mL, *P* = 0.32). In both groups, all patients were discharged on foot, and the medications at discharge did not differ between the two groups (Table 1). During a mean follow-up period of 851 ± 366 (median 776 [667–1181] days), 1 (14%) patient in the Impella group experienced a major bleeding event (gastrointestinal bleeding) at 450 days after discharge; 3 (17%) non-Impella patients were hospitalized due to heart failure at 8, 95, and 218 day, respectively.

### Subanalysis of the baseline characteristics and CMRI assessment between the Impella group and non-Impella groups who did and did not experience cardiogenic shock.

The patients in the non-Impella group were divided according to the presence or absence of cardiogenic shock and were compared with the Impella group. The BMI, systolic blood pressure and minimum blood pressure from admission to the PCI, dyslipidemia rate, and use of IAPB differed significantly between the Impella and non-Impella groups with and without cardiogenic shock. There were no differences in the other baseline clinical characteristics including the age, sex, coronary artery regions, and transthoracic echocardiographic parameters on admission between the Impella and non-Impella groups with and without cardiogenic shock (Additional file 1: Table S1). CMRI in the subacute phase revealed that the LVEF significantly increased, and the LVEDV and LVESV decreased in a stepwise fashion from the non-Impella with cardiogenic shock group, to the Impella without cardiogenic shock group, and to the Impella group (LVEF: 28 ± 5.8, 37 ± 6.6, and 45 ± 12%, respectively, *P* = 0.018; LVEDV: 207 ± 28, 178 ± 41, and 143 ± 27 mL, respectively, *P* = 0.011; LVESV: 149 ± 33, 111 ± 37, and 79 ± 26 mL, respectively, *P* = 0.011). The GLS did not differ among the 3 groups, but the GRS and GCS increased significantly in a stepwise fashion from the non-Impella with cardiogenic shock group, to the Impella without cardiogenic shock group, and to the Impella group (GRS: 11 ± 2.9, 16 ± 5.0 and 23 ± 9.1%, respectively, *P* = 0.050; GCS: − 7.0 ± 1.9, − 10 ± 3.0 and − 13 ± 4.3%, respectively, *P* = 0.018). The global systolic and diastolic SRs did not differ in the longitudinal axis, while the global systolic and diastolic SRs in the radial and circumferential axes increased gradually from the non-Impella with cardiogenic shock group, to the Impella without cardiogenic shock group, and to the Impella group, and those differences were more significant in the non-infarcted areas than infarcted areas (Additional file 1: Table S2).

## Discussion

This study had two major findings: (1) with similar baseline patient characteristics on admission, the LVEF identified on CMRI 19.8-days later was significantly greater and the LVEDV and LVESV smaller, while the GRS and GCS rather than the GLS, and systolic and diastolic SRs in the radial and circumferential axes rather than in the longitudinal axis were significantly greater in the Impella group than non-Impella group, despite no difference in the infarct size, (2) a greater peak strain and the systolic and diastolic SRs in the Impella rather than non-Impella group were more predominant in the non-infarcted areas than infarcted areas, and (3) there was no difference in the length of the hospital stay between the two groups. All patients were discharged on foot; 3 patients in the non-Impella group required a re-admission due to heart failure, while no patients experienced heart failure in the Impella group.

### Effect of the Impella on the prevention of LV remodeling

It is widely known that shortening the DTBT enables a reduction in the infarct size; however, the use of an Impella has not yet been demonstrated to reduce the infarct size in humans. When assessed via CMRI, our study showed no reduction in the infarct size in the Impella group when compared with the non-Impella group. Despite the similar transthoracic echocardiographic LVEF, LVEDV, and LVESV on admission between the two groups, CMRI in the subacute phase revealed that the LVEF was significantly greater and LVEDV and LVESV smaller by LV unloading in the Impella group. In contrast, a canine model study reported by Saku et al. showed that 4 weeks after ischemia/reperfusion with an Impella the infarct size was strikingly reduced by > 80%, but similar to our results, it preserved the echocardiographic LVEF and reduced the LVESV relative to that when not using the Impella [16]. This study further provided a new insight into the detailed LV function according to the peak strains and systolic and diastolic SRs segmented by the global LV and infarcted and non-infarcted areas. Myocardial strain is an acute indicator of the contractile force, which can usually be calculated in the circumferential, longitudinal, and radial axes of the myocardial contraction. The SR is the change in the strain for a given vector as a function of time [17]. The GCS and diastolic SRs are reported to be objective, sensitive markers of the myocardial systolic and diastolic function [18]. Another report demonstrated that both the longitudinal and circumferential systolic SRs were independent predictors of the outcomes after an MI, whereas only the circumferential systolic SR was predictive of remodeling. The data suggested that a preserved circumferential function might serve to restrain ventricular enlargement after an MI [19]. Our MRI data also showed that the peak strain and systolic and diastolic SRs in the circumferential and radial axes were significantly greater in the Impella than non-Impella group. A subanalysis revealed that, although the baseline characteristics in the Impella patients were similar to those in the non-Impella patients with cardiogenic shock and might be slightly worse than that in the non-Impella patients without cardiogenic shock, the LVEF, GRS, and GCS were the greatest and LVEDV and LVESV the smallest in the Impella group as compared to the other two groups. Importantly, although the Impella group had slightly greater radial and circumferential systolic and diastolic SRs in the infarcted area, the greatest magnitudes of the peak strain and systolic and diastolic SRs were in particular in the circumferential axis and non-infarcted areas. A pathological study reported that longitudinal fibers are present in the endocardium, while circumferential fibers predominate in the mid-wall of the myocardium. In the subepicardial layer the fibers are longitudinally oriented but directed from the apex to the base. Due to this complex architecture, during systole, the LV deforms along different directions determining the longitudinal and circumferential shortening, radial thickening, and torsion [20]. Therefore, our findings suggested that the Impella would slightly provide a favorable impact on the systolic and diastolic function in the infarcted area, but a more significant impact in the non-infarcted mid-walls of the myocardium (circumferential axis fibers) by unloading the entire LV. That preserved systolic and diastolic strain function in global areas provided by the Impella resulted in a greater LVEF and reduced LVEDV and LVESV [21]. Bulluck et al. [22] demonstrated that an increased extracellular volume fraction of the remote myocardium acutely, was maintained higher at 5 ± 2 months after a STEMI in patients who developed adverse LV remodeling (defined as a ≥ 20% increase in the LV end diastolic volume) than in those without remodeling. Tsuda et al. [23] showed there is molecular and immune-histochemical evidence of interstitial fibrosis in the remote myocardium as early as 72 h post-MI. Volders et al. [24] indicated postmortem histological evidence of an increase in the interstitial collagen in the remote myocardium of infarcted patients when compared to control patients. Moreover, remote zone non-contrast T1-mapping provided independent and incremental prognostic information about the clinical risk factors and traditional CMRI outcome markers in STEMI patients treated by a primary PCI [25]. The mechanism for improving the non-infarct area by the Impella is incompletely known; however, recent findings have indicated that post-MI LV remodeling in the subacute phase is a multifactorial process that may involve excessive inflammation and/or subtle fibrosis of the remote (non-infarct) myocardium, and the progression may be protected by the Impella.

### Outcomes after Impella placement

Recent randomized control trials or observation studies have shown controversial results regarding the prognostic effect of the Impella. The small IMPRESS in Severe SHOCK trial (n = 48) did not show any beneficial effects of the Impella CP as compared to IABP [9]. A recent matched-pair analysis (237 matched pairs) comparing Impella- and IABP-treated CS patients, showed a similar 30-day mortality in both groups [26]. The lactate level was lower and baseline LVEF assessed by transthoracic echocardiography higher in our Impella patients than in the previous reports, suggesting a better condition in our study patients, possibly due to the early DTBT time in this study. Furthermore, the Impella was placed before the PCI in all study patients, resulting in a better cardiac condition, i.e., the GRS, GSC, and systolic and diastolic SRs improved particularly in the non-infarcted area, and the LV chamber size became reduced. It is known that an increase in the LVEDV is an important risk for heart failure [22, 25]. The LVESV and LV mass have also been reported to be predictive factors for heart accidents [27, 28]. Despite no difference in the NT-proBNP, LGE, and length of the hospital stay at discharge between the patients with and without an Impella, myocardial protective effects via early unloading by the Impella as seen with our patients may lead to better clinical outcomes. This was implied by our results in which 3 patients (17%) in the non-Impella group suffered from rehospitalizations due to heart failure, while none in the Impella group suffered any heart failure events. The STEMI-DTU Trial (NCT03947619) is currently ongoing in the United States; the purpose of that study is to evaluate whether using the Impella CP System for 30 min prior to catheterization can reduce the damage to the heart caused by a heart attack, as compared to the current standard of care. This trial would answer our question whether early unloading by the Impella would improve the clinical outcomes in MI patients with cardiogenic shock.

### Study limitations

First, our study was limited by the retrospective design of our analysis. In particular, the decision of whether to use an Impella, IABP, or VA-ECMO was dependent on the physicians’ discretion and the time period when the Impella could be used in Japan. An IABP is generally placed in MI patients without any severe risk, so a healthier bias in the non-Impella group might have persisted. To minimize that effect, we conducted a subanalysis after dividing the non-Impella group patients into the presence or absence of cardiogenic shock. A preserved LV systolic and diastolic function and the smallest LV chambers were persistently observed in the Impella group than other two non-Impella groups. The prevention of LV remodeling by the Impella might have been reasonable. Second, our data was the result of a single-center registry providing a limited number of patients with a broad anterior STEMI. This may have failed to gain a statistical significance in some parameters. Finally, the follow-up period was too short to obtain a statistical difference in the clinical outcomes between the Impella and non-Impella groups. Further larger studies over a longer period are required to identify the prognostic effect of the Impella.

## Conclusions

Early implantations of the Impella before the PCI preserved the progression of a reduced CMRI-derived LVEF and enlargement of the LVEDV and LVESV in the subacute phase. The CMRI strain analysis revealed greater values of the peak strains and systolic and diastolic SRs in the Impella group than non-Impella group, and those parameter-differences were predominant in the circumferential axis and non-infarcted areas. This study provided mechanistic insight into understanding the usefulness of the Impella for cardioprotective effects to prevent LV remodeling.

## Supplementary Information

**Additional file 1**. Baseline characteristics and CMRI strain parameters between the Impella, non-Impella with cardiogenic shock, and non-Impella without cardiogenic shock groups.

## Data Availability

The datasets used and/or analyzed during the current study are available from the corresponding author on reasonable request.

## Abbreviations

AMI: Acute myocardial infarction
BMI: Body mass index
CMRI: Cardiac magnetic resonance image
DTBT: Door-to-balloon time
eGFR: Estimated glomerular filtration rate
IABP: Intra-aortic balloon pumping
LAD: Left anterior descending
LGE: Late gadolinium enhancement
LV: Left ventricular
LVEDV: Left ventricular end-diastole volume
LVEF: Left ventricular ejection fraction
LVESV: Left ventricular end-systolic volume
MI: Myocardial infarction
SSFP: Standard steady-state free precession
NT-proBNP: N-terminal pro-brain natriuretic peptide
CI: Percutaneous coronary intervention
SR: Systolic strain rate
STEMI: ST-elevation myocardial infarction
VA-ECMO: Veno-arterial extracorporeal membrane oxygenation
