# Supplementary material for: Protective effect of the Impella on the left ventricular function after acute broad anterior wall ST elevation myocardial infarctions with cardiogenic shock: cardiovascular magnetic resonance imaging strain analysis
Source: BMC Cardiovasc Disord. 2022 Apr 28;22:201. doi: 10.1186/s12872-022-02632-7 (PMC9052554; doi:10.1186/s12872-022-02632-7)
Supplement: Supplementary file 1 — Additional file 1. Baseline characteristics and CMRI strain parameters between the Impella, non-Impella with cardiogenic shock, and non-Impella without cardiogenic shock groups. [file 12872_2022_2632_MOESM1_ESM.docx]

**Table S1. Baseline characteristics between the Impella, non-Impella with cardiogenic shock, and non-Impella without cardiogenic shock groups**

|  | Impella  (n=7) | Non-Impella  (n=18) | | P-Value |
| --- | --- | --- | --- | --- |
|  |  | CS (+) (n=5) | CS (-) (n=13) |  |
| Age, years | 67±10 | 54±4 | 61±15 | 0.14 |
| Male gender | 6 (85) | 5 (100) | 12 (92) | 0.66 |
| BMI (m^2^/kg) | 21±1.5 | 24±2.7 | 24±4.1 | 0.050 |
| Hemodynamic variables on admission |  |  |  |  |
| Heart rate (beats/min) | 85±18 | 87±31 | 88±14 | 0.88 |
| Systolic blood pressure (mmHg) | 97±25 | 80±54 | 131±13^∗^ | 0.027 |
| Diastolic blood pressure (mmHg) | 77±19 | 78±49 | 86±14 | 0.64 |
| Minimum systolic blood pressure from admission to PCI | 91±20 | 81±48 | 116±13^∗^ | 0.022 |
| Cardiogenic Shock | 7 (100) | 5 (100) | 0 (0) | <0.001 |
| History or comorbidities |  |  |  |  |
| Current smoking | 1 (17) | 3 (75) | 6 (42) | 0.18 |
| Hypertension | 5 (71) | 4 (80) | 8 (62) | 0.88 |
| Diabetes Mellitus | 1 (14) | 2 (40) | 3 (23) | 0.59 |
| Dyslipidaemia | 7 (100) | 3 (60) | 5 (39) | 0.028 |
| Blood values on admission |  |  |  |  |
| Lactate (mmol/l) | 2.9±1.4 | 2.9±1.1 | 2.1±1.0 | 0.27 |
| Hb (mg/dl) | 13.4±1.6 | 14.3±0.8 | 15.0±1.2 | 0.08 |
| eGFR (mL/min/1.73m^2^) | 65±16 | 66±23 | 77±20 | 0.33 |
| Blood values at discharge |  |  |  |  |
| NT-proBNP at discharge (pg/ml) | 922 (130,1643) | 1662 (1094,2821) | 831  (334,2343) | 0.39 |
| Support device |  |  |  |  |
| Mechanical ventilation | 2 (29) | 1 (20) | 2 (15) | 0.78 |
| IABP | 0 (0) | 4 (80) | 8 (62) | 0.009 |
| VA-ECMO | 1 (14) | 1 (20) | 0 (0) | 0.29 |
| Anterior STEMI | 6 (100) | 5 (100) | 6 (100) |  |
| Infarct-related artery |  |  |  |  |
| Left main stem | 1 (17) | 0 (0) | 0 (0) |  |
| Left anterior descending | 6 (83) | 5 (100) | 12 (100) | 0.67 |
| Multivessel disease | 1 (17) | 1 (20) | 0 (0) | 0.06 |
| Stent placement |  |  |  |  |
| Drug-eluting stent | 7 (100) | 5 (100) | 13 (100) | 0.59 |
| Number of DES stents | 1.2±0.4 | 1.0±0 | 1.2±0.4 | 0.34 |
|  |  |  |  |  |
| Initial TIMI flow 0 | 4 (57) | 5 (100) | 6 (46) | 0.15 |
| Final TIMI flow 3 | 7 (100) | 5 (100) | 12 (92) | 0.62 |
| Max CK (IU/L) | 7058±4994 | 10503±6539 | 5454±3002 | 0.25 |
|  | 5964 (2262,11273) | 9302 (7014,15972) | 5232  (2941,6891) |  |
| Max CK MB (IU/L) | 527±355 | 775±583 | 448±271 | 0.56 |
|  | 542 (168,920) | 754 (412,1330) | 386 (281,594) |  |
| Door to balloon time (min) | 60±16 | 47±21 | 58±25 | 0.43 |
| Echo parameter on admission |  |  |  |  |
| LVEF (%) | 49±11 | 44±2.4 | 50±8 | 0.51 |
| LVEDV (mL) | 107±15 | 127±29 | 115±34 | 0.65 |
| LVESV (mL) | 50±17 | 64±21 | 55±19 | 0.52 |
| E (cm/sec) | 73±12 | 53±15^∗^ | 68±22 | 0.15 |
| A (cm/sec) | 73±21 | 63±16 | 70±25 | 0.66 |
| E/A | 1.1±0.5 | 0.9±0.3 | 1.2±0.7 | 0.39 |
| Duration of IMPELLA support (days) | 4.0±1.7 |  |  |  |
| Medications at discharge |  |  |  |  |
| DAPT | 7 (100) | 5 (100) | 13(100) |  |
| RAS inhibitor | 7 (100) | 5 (100) | 13 (100) |  |
| Beta blocker | 7 (100) | 5 (100) | 12 (92) | 0.67 |
| Statin | 7 (100) | 5(100) | 13 (100) |  |
| Days of coronary care unit (days) | 7.4±3.7 | 8.8±4.5 | 5.8±3.8 | 0.42 |
| Days of hospital admission (days) | 27±8 | 30±8 | 24±8 | 0.42 |
| Days of CMRI after admission (days) | 16±7 | 26±98 | 17±9 | 0.07 |

Values are shown as the number (%), mean±SD, or median (interquartile ranges). BMI, body mass index; CMRI, cardiac magnetic resonance image; CS, cardiac shock; CK, creatinine kinase; DES, drug eluting stent; DAPT, dual antiplatelet therapy; eGFR, estimated glomerular filtration rate; Hb, haemoglobin; IABP, intra-aortic balloon pumping; LVEF, left ventricular ejection fraction; LVEDV, left ventricular end-diastole volume; LVESV, left ventricular end-systolic volume; NT-pro BNP, n-terminal pro brain natriuretic peptide; PCI, percutaneous coronary intervention; RAS, renin angiotensin system; STEMI, ST-elevation myocardial infarction; TIMI, thrombolysis in myocardial infarction; VA-ECMO, veno-arterial extracorporeal membrane oxygenation. P values are determined by a Kruskal-Wallis test followed by the post hoc Bonferroni corrected Mann-Whitney U post-hoc analysis or chi-square text. ∗P<0.05 vs. Impella group.

**Table S2. CMRI strain parameters between the Impella, non-Impella with cardiogenic shock, and non-Impella without cardiogenic shock groups**

| CMRI strain parameters | Impella  (n=7) | Non-Impella  (n=18) | | P-Value |
| --- | --- | --- | --- | --- |
|  |  | CS (+) (n=5) | CS (-) (n=13) |  |
| LVEF (%) | 45±12 | 28±5.8^∗^ | 37±6.6 | 0.018 |
| LVEDV (mL) | 143±27 | 207±28^∗^ | 178±41 | 0.011 |
| LVESV (mL) | 79±26 | 149±33^∗^ | 111±37 | 0.011 |
| LV Mass (g) | 82±13 | 99±18 | 97±24 | 0.14 |
| Infarcted size  (% of LV myocardial mass) | 29±13 | 36±11 | 29±11 | 0.44 |
| LV longitudinal |  |  |  |  |
| Global |  |  |  |  |
| Peak Strain (%) | -9.3±2.0 | -6.9±3.3 | -8.5±1.5 | 0.49 |
| Systolic strain rate (1/s) | -0.8±0.2 | -0.3±0.7 | -0.5±0.4 | 0.07 |
| Diastolic strain rate (1/s) | 0.7±0.2 | 0.5±0.3 | 0.4±0.3 | 0.27 |
| Infarcted area |  |  |  |  |
| Peak Strain (%) | -6.6±2.3 | -5.2±4.5 | -6.2±3.0 | 0.94 |
| Systolic strain rate (1/s) | -0.5±0.4 | -0.2±0.6 | -0.3±0.4 | 0.53 |
| Diastolic strain rate (1/s) | 0.3±0.3 | 0.3±0.1 | 0.3±0.3 | 0.97 |
| Non-Infarcted area |  |  |  |  |
| Peak Strain (%) | -13±3.0 | -9.0±6.9 | -12±1.7 | 0.54 |
| Systolic strain rate (1/s) | -1.2±0.5 | -0.4±0.5 | -0.7±0.5 | 0.27 |
| Diastolic strain rate (1/s) | 1.2±0.4 | 0.7±0.6 | 0.6±0.5 | 0.08 |
| LV radial |  |  |  |  |
| Global |  |  |  |  |
| Peak Strain (%) | 23±9.1 | 11±2.9 | 16±5.0 | 0.050 |
| Systolic strain rate (1/s) | 1.4±0.8 | 0.6±0.2^∗^ | 0.9±0.3 | 0.035 |
| Diastolic strain rate (1/s) | -1.3±0.5 | -0.4±0.3^∗^ | -0.7±0.3 | 0.012 |
| Infarcted area |  |  |  |  |
| Peak Strain (%) | 19±9.9 | 8.2±2.2 | 13±7.5 | 0.10 |
| Systolic strain rate (1/s) | 1.2±0.8 | 0.4±0.2^∗^ | 0.7±0.4 | 0.037 |
| Diastolic strain rate (1/s) | -0.9±0.6 | -0.1±0.4 | -0.5±0.5 | 0.078 |
| Non-Infarcted area |  |  |  |  |
| Peak Strain (%) | 27±8.2 | 14±4.2^∗^ | 19±6.7 | 0.041 |
| Systolic strain rate (1/s) | 1.7±0.7 | 0.9±0.2^∗^ | 1.1±0.4 | 0.039 |
| Diastolic strain rate (1/s) | -1.9±0.4 | -0.6±0.2^∗^ | -0.8±0.3^∗^ | 0.004 |
| LV circumferential |  |  |  |  |
| Global |  |  |  |  |
| Peak Strain (%) | -13±4.3 | -7.0±1.9^∗^ | -10±3.0 | 0.018 |
| Systolic strain rate (1/s) | -0.9±0.4 | -0.4±0.1^∗^ | -0.7±0.2 | 0.024 |
| Diastolic strain rate (1/s) | 0.8±0.2 | 0.2±0.3^∗^ | 0.5±0.3^∗^ | 0.005 |
| Infarcted area |  |  |  |  |
| Peak Strain (%) | -12±5.5 | -4.7±2.0 | -8.8±4.7 | 0.081 |
| Systolic strain rate (1/s) | -0.8±0.4 | -0.2±0.1^∗^ | -0.5±0.3 | 0.036 |
| Diastolic strain rate (1/s) | 0.6±0.3 | -0.02±0.3^∗^ | 0.3±0.3 | 0.031 |
| Non-Infarcted area |  |  |  |  |
| Peak Strain (%) | -15±3.0 | -10±1.8^∗^ | -12±3.4 | 0.020 |
| Systolic strain rate (1/s) | -1.1±0.4 | -0.6±0.2 | -0.8±0.2 | 0.053 |
| Diastolic strain rate (1/s) | 1.0±0.1 | 0.5±0.2^∗^ | 0.6±0.2^∗^ | 0.002 |

CMRI, cardiac magnetic resonance image; LV, left ventricular; LVEF, left ventricular ejection fraction; LVEDV, left ventricular end-diastole volume; LVESV, left ventricular end-systolic volume. P values are determined by a Kruskal-Wallis test followed by the post hoc Bonferroni corrected Mann-Whitney U post-hoc analysis ∗P<0.05 vs. Impella group.
